# Identification of Heat Responsive Genes in *Brassica napus* Siliques at the Seed-Filling Stage through Transcriptional Profiling

**DOI:** 10.1371/journal.pone.0101914

**Published:** 2014-07-11

**Authors:** Erru Yu, Chuchuan Fan, Qingyong Yang, Xiaodong Li, Bingxi Wan, Yanni Dong, Xuemin Wang, Yongming Zhou

**Affiliations:** 1 National Key Laboratory of Crop Genetic Improvement, Huazhong Agricultural University, Wuhan, Hubei, China; 2 Guizhou Institute of Prataculture, Guizhou Academy of Agricultural Sciences, Guiyang, Guizhou, China; 3 Department of Biology, University of Missouri, Saint Louis, Missouri, United States of America; Universidad Miguel Hernández de Elche, Spain

## Abstract

High temperature stress results in yield loss and alterations to seed composition during seed filling in oilseed rape (*Brassica napus*). However, the mechanism underlying this heat response is poorly understood. In this study, global transcription profiles of 20 d-old siliques of *B. napus* were analyzed after heat stress using a Brassica 95k EST microarray. The up-regulated genes included many *HSF*/*HSP* transcripts and other heat-related marker genes, such as *ROF2*, *DREB2a*, *MBF1c* and *Hsa32*, reflecting the conservation of key heat resistance factors among plants. Other up-regulated genes were preferentially expressed in heat-stressed silique walls or seeds, including some transcription factors and potential developmental regulators. In contrast, down-regulated genes differed between the silique wall and seeds and were largely tied to the biological functions of each tissue, such as glucosinolate metabolism in the silique wall and flavonoid synthesis in seeds. Additionally, a large proportion (one-third) of these differentially expressed genes had unknown functions. Based on these gene expression profiles, *Arabidopsis* mutants for eight heat-induced *Brassica* homologous genes were treated with different heat stress methods, and thermotolerance varied with each mutation, heat stress regimen and plant development stage. At least two of the eight mutants exhibited sensitivity to the heat treatments, suggesting the importance of the respective genes in responding to heat stress. In summary, this study elucidated the molecular bases of the heat responses in siliques during later reproductive stages and provides valuable information and gene resources for the genetic improvement of heat tolerance in oilseed rape breeding.

## Introduction

High temperature is a major abiotic stress that severely restricts crop production [1]. Impaired fertility and yield loss due to heat stress are widely reported for various crops, including wheat [2], rice [1], corn [3] and cotton [4]. With global warming growing more severe, the risk of high temperature effects on crop production is increasing.

Oilseed rape (*Brassica napus*) is the third largest oilseed crop in the world [5]. As a cool season crop, oilseed rape and other brassica oil-producing species are sensitive to heat stress. Comprehensive analyses of weather and long-term yield records reveal that high temperatures impose harmful effects on oilseed rape yields worldwide [6], [7]. Two *B. napus* cultivars, Delta and Westar, were shown to be almost completely sterile when grown with a temperature regimen of 27/17°C (day/night) [8]. During the period from bolting to the end of flowering, a threshold temperature of 29.5°C resulted in seed yield loss in three different *Brassica* species: *B. napus*, *B. rapa* and *B. juncea* [9]. Short periods of heat stress are also destructive to plants, particularly when they occur at critical developmental stages. For many crops, reproduction is the most heat-sensitive stage, and heat stresses at this stage result in more severe reduction of yields [1]. In the oilseed rape cultivar Zollerngold, it was found that 75% of pods was developed from flowers opened within the first two weeks [10]. Incubation at high temperature of 35/15°C (day/night) for 7 d during this stage reduces seed yields by 89% on the main stems, and on average, seed yield per plant decreases to 52% in three tested *Brassica* species [11].

Seed storage components, including oil and proteins, accumulate between 2 and 5 weeks after flowering in oilseed rape [12]. When a heat stress of 35/18°C (day/night) was applied to three *B. juncea* cultivars and one *B. napus* cultivar for 10 d at three distinct reproductive stages, the seed yield per plant was reduced by 77% at the pod development stage, 58% at the flowering stage and 15% at the bud formation stage [13]. A moderate heat treatment of 28/23°C (day/night) on *B. napus* for 10 d from 20 to 30 days after flowering (DAF) was shown to lead to an altered fatty acid profile, including increased oleic acid and reduced linoleic and linolenic acids [14]. A very high temperature stress of 38/23°C (day/night) applied for 5 d from 25 to 29 DAF moderately affected fatty acid composition by decreasing the oil content and reduced the seed yield by 52% on main stems, due to the reduction of seed weight [14].

Previous studies in *Brassica* species have demonstrated a negative relationship between high temperature stress and seed yield and quality, but the mechanisms underlying these phenomena are largely unknown. In the model plant *Arabidopsis*, heat transcription factors (*HSFs*) regulate a subset of heat-responsive genes including heat shock proteins (*HSPs*), which is responsible for basal plant thermotolerance during the vegetative growth stage [15], [16]. In *B. rapa* (ssp. *perkinensis* cv. Jangwon), the expression profile of 7-d-old seedlings exposed to heat stress over a time course from 0.25 to 12 h demonstrated that heat treatment results in the significant accumulation of *HSFs* and *HSPs* over time, and some cell wall-modifying genes are up-regulated, particularly at later time points [17].

MicroRNAs (miRNAs) may also participate in heat resistance in 3-week-old plants of *B. rapa* (ssp. *chinensis*); five miRNA families conserved within *Arabidopsis thaliana* and another four novel miRNA families are responsive to heat stress, as was revealed by sequencing [18]. These data provide a foundation to explore the molecular basis of thermotolerance in *B. napus* during the vegetative period. During the reproductive stage, at least one *HSP* transcript is induced in *B. napus* pollen and pistil more dramatic than in leaves, suggesting that developing gametophytes are responsive to high temperature stress [19]. Gene expression profiles of male reproductive organs after heat stress have been investigated in rice [20], barley [21] and tomato [22]. In these tissues, the transcription levels of *HSFs* and *HSPs* are elevated; in contrast, genes involved in cell proliferation and DNA replication as well as genes encoding hydrolytic enzymes, which are predominantly expressed in tapetum cells, are silenced after heat stress during pollen development (reviewed in [23]).

Seeds are the harvested organs of *B. napus.* The production of seed storage substances (mainly oil and starch) during seed filling is affected by photosynthetic activities in the silique wall (SW) rather than in leaves [24]. However, little is known about the similarities and differences between the heat-responsive patterns in the SW and seeds during pod development. In this study, we employed a microarray analysis with SW and seeds from 20 DAF siliques of *B. napus* that had undergone heat stress. We identified different sets of genes that were stimulated dramatically in response to heat stress in both the SW and seeds. In addition to notable changes in the expression of *HSFs/HSPs* and other signature heat-responsive genes, many other responsive genes exhibited organ-specific expression. We also identified the pathways that were regulated in two organs in response to heat stress in *B. napus*. Our data provide a basic understanding of the heat responses in developing siliques at transcriptional levels. Furthermore, we used functional genomic tools in *Arabidopsis* to identify novel genes involved in the heat response.

## Results and Discussion

### Overview of gene expression affected by heat stress in *B. napus* siliques at the seed-filling stage

To identify genes responsive to heat stress in oilseed rape at the seed-filling stage, a Brassica 95k EST microarray (jointly developed by the John Innes Centre and Cogenics [25]) was used to profile the transcripts from both the SW and seeds separated from 20 DAF siliques. To mimic temperature changes in the field, the temperature was increased linearly from 23°C to 35°C over 4 h, and after heat stress for 5 h at 35°C, the temperature was reduced to 23°C at the same rate (Figure S1). Samples were taken for array analysis at 24 h and 48 h after heat stress.

**Figure 1:**
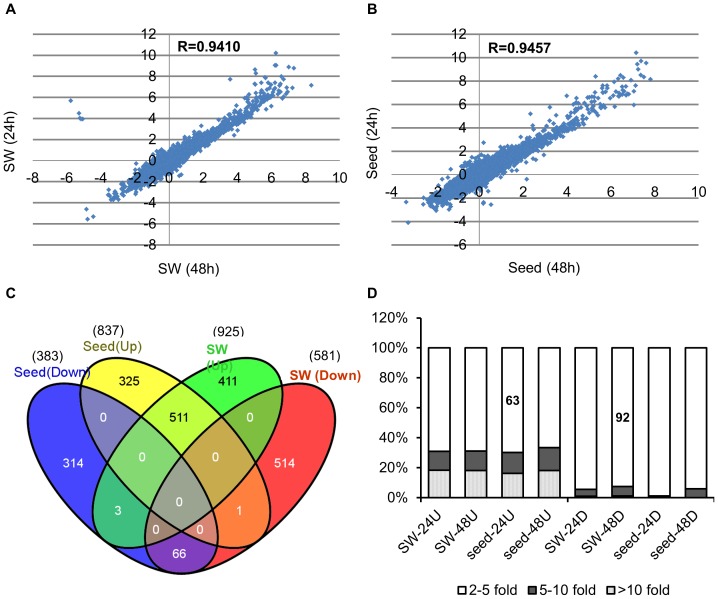
Expression characteristics of heat-stressed siliques. Correlation analysis of the SW (A) and seed (B) transcriptomes after 24 h and 48 h recovery times. (C) Venn diagrams showing the intersection of differentially expressed transcripts in the heat-stressed SW and seed. (D) Comparison of increased or decreased transcripts in different samples. Abbreviations: 24 U/48 U and 24 D/48 D indicate up-regulated and down-regulated genes after the 24 h/48 h recovery times either in the SW or seeds.

To reduce false positive results, a stringent filtration protocol was used to analyze the microarray data (see Materials and methods). Only probe sets with the consensus detection call PP at both sampling time points (24 h and 48 h) were considered to be positive for mRNA. After filtration, 19,263 of the 78,278 unique genes on the arrays were included in subsequent analyses. The Pearson correlation coefficients of the ratio of genes expressed under heat stress to those under normal conditions between 24 h and 48 h were 0.94 in the SW and 0.95 in seeds (Figures 1A-1B). Because most expressed genes exhibited similar expression patterns after 24 h and 48 h of recovery time for both organs, the differentially expressed genes responsive to heat stress were characterized as those exhibiting similar changes at both sampling points.

After heat stress, 925 and 837 genes were up-regulated by more than 2-fold in the SW and seeds, respectively, compared with the control (Figure 1C), while about half as many genes were down-regulated in both organs (SW: 581 genes, seeds: 383 genes). The ratio of up-regulated to down-regulated genes was consistent with a previous report of *B. rapa* [17] but differed from *Arabidopsis*, for which the ratio was 1∶1.6 [25]. Collectively, 1,248 up-regulated and 898 down-regulated genes were detected in heat-stressed siliques (SW plus seeds) (Figure 1C), accounting for 11.1% of the total expressed genes. This proportion was similar to results in *Arabidopsis* [26] and Chinese cabbage [17]. In addition, up-regulated transcripts demonstrated greater relative changes than down-regulated genes in our study. More than 92% of down-regulated genes exhibited changes in expression of less than 5-fold, while more than 37% of up-regulated genes exhibited over 5-fold increased expression (Figure 1D). Thus, considerable numbers of genes were reprogrammed and primarily enhanced due to heat stress, and the activation of transcripts in both the SW and seeds might be fundamental to basal thermotolerance in *B. napus*.

### Large sets of stress-related genes were significantly up-regulated in both the SW and seeds upon heat stimulation

**Figure 2:**
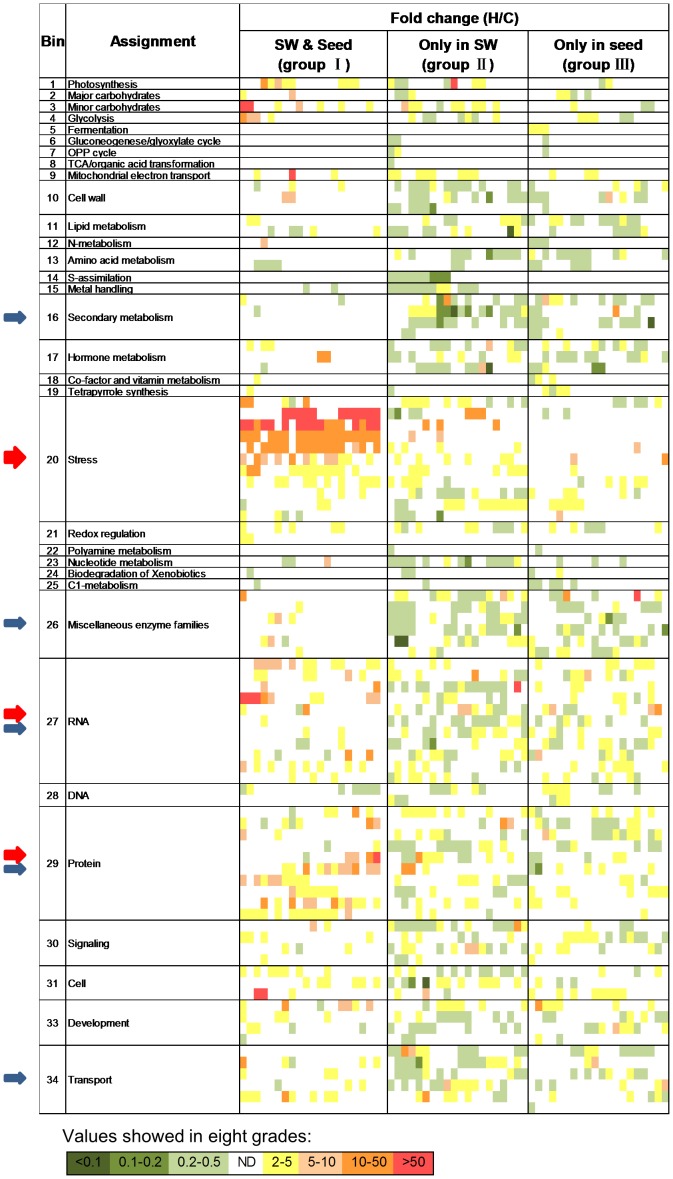
Heat map of the differentially expressed genes in 34 functional categories. Fold change was expressed as the ratio of normalized expression of heat to control, and the value was divided into eight grades as shown in the diagram. ND designates no differentially expressed unique genes. Arrows in red/blue highlight the categories with abundant up-regulated/down-regulated genes. The width of the arrow is proportional to the number of up-regulated/down-regulated genes. The corresponding genes in groups I/II/III of the heat map are listed in Table S2.

**Figure 3:**
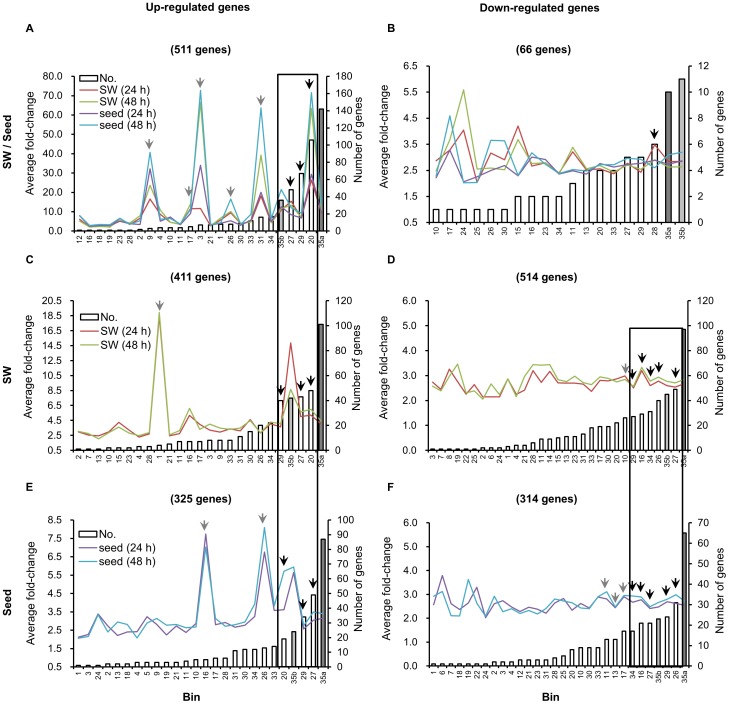
Statistical analysis of functional classifications for differentially expressed genes with different patterns. A-F show the average fold-change and abundance in different functional categories of up-regulated genes in group I (A), group II (C) and group III (E) and the same parameters for the down-regulated genes in group I (B), group II (D) and group III (F). Dark-gray and light-gray bars represent the genes with unknown functions that were conserved (Bin 35a) and not conserved (Bin 35b), respectively, with *Arabidopsis*. Arrows in black indicate the important functional categories that had the most abundant genes (usually with large alterations); arrows in gray indicate the important functional categories that had less abundant genes but usually contained genes that had been dramatically altered or abundant moderately altered genes. The two large open boxes in A-F indicate the common categories in corresponding samples.

All differentially expressed genes were classified into three groups: genes expressed in both the SW and seeds (group I), genes expressed only in the SW (group II) or genes expressed only in seeds (group III) (Figure 2). Using MapMan software (v. 3.5.1), the differentially expressed genes were functionally designated into 35 categories (Bins) in addition to a category for non-assigned functions (Figures 3A-3F and Table S1).

In total, 40.9% (511) of the up-regulated genes were present in group I (Figure 1C). These genes were functionally classified into 23 categories, with the average fold-change ranging from 2.1 to 72.8 in addition to uncharacterized genes, which were documented separately (Figures 2, 3A and Table S1). The most abundantly up-regulated genes were related to stress (106 genes, 20.7%, Bin 20), protein (67, 13.1%, Bin 29) and RNA (48, 9.4%, Bin 27) (Figures 2, 3A and Table S1).

In contrast to the substantial overlap of up-regulated genes in the SW and seeds, only 66 genes were down-regulated in both organs after heat stress (Figure 1C). These genes were classified into 23 categories with the average fold-change ranging from 2.0 to 5.6 (Figures 2, 3B and Table S1). The most abundant category (6 genes) was related to DNA repair (Bin 28, Figure 3B and Table S1). In *Arabidopsis*, disruption of a putative DNA helicase required for nucleotide excision repair results in the phenotype mimicking the wild type (WT) undergone moderate heat stress [27], indicating a connection between DNA repair and the heat stress response.

### A subset of genes was involved in basal heat-shock responses

In agreement with previous studies [26], [28], the expression of 13 *HSFs* and 91 *HSPs*, belonging to 6 *Hsf* sub-families (*A2*, *A7a*, *B1*, *B2a* and *B2b*) and 6 *Hsp* families (*DnaJ/Hsp40*, *Hsp60/10*, *Hsp70*, *Hsp90*, *Hsp101* and *sHsp*), respectively, were strikingly up-regulated by an average of 36.5-fold (Tables S3, S4), suggesting commonalities in the heat stress response in these two organs. In addition to the *HSP* genes, *BAG6* transcripts (EE519954, JCVI_15005, EV035159, EL590013 and JCVI_25068) were notably up-regulated by 80-fold in both organs (Tables 1, S3). This gene is heat-induced and regulated by *HsfA2* in *Arabidopsis* [29], [30]. Other strongly induced stress-responsive genes with average changes of more than 10-fold included *HOP3* (JCVI_16615 and JCVI_15301), a putative co-chaperone of Hsp90/Hsp70 [31], *BI-1* (JCVI_1535), which regulates heat-shock-induced cell death [32], and *Hsa32* (JCVI_13423), an essential heat tolerance factor [33] (Tables 1, S3).

**Table 1:** List of genes (*Hsf* and *Hsp* genes excluded) exhibiting more than 10-fold changes in expression in both the SW and seeds (Group I).

Gene	Annotation	Bin	ATH^a^	Gene name	FC^b^	Reference
JCVI_400	Oxygen-evolving enhancer protein 2-1, chloroplast precursor	1	AT1G06680	*OE23*	13.98	
JCVI_26215	Galactinol synthase	3	AT2G47180	*GolS1*	163.71	[47]
JCVI_40980	Galactinol synthase	3	AT2G47180	*GolS1*	59.62	[47]
EE423714	Fructose-bisphosphate aldolase	4	AT2G36460	*FBA6*	10.12	[45]
JCVI_33865	Protein At5g25450	9	AT5G25450	*Cytochrome bd ubiquinol oxidase*	71.54	
JCVI_802	Ethylene-responsive transcriptional coactivator-like protein	17	AT3G24500	*MBF1c*	25.49	[48]
JCVI_27680	Probable pyridoxal biosynthesis protein PDX1.2	17	AT3G16050	*PDX1.2*	19.74	
JCVI_15005	Putative At2g46240	20	AT2G46240	*BAG6*	123.70	[29], [30]
EE519954	Putative At2g46240	20	AT2G46240	*BAG6*	115.76	[29], [30]
EL590013	Putative At2g46240	20	AT2G46240	*BAG6*	106.10	[29], [30]
EV035159	Putative At2g46240	20	AT2G46240	*BAG6*	95.94	[29], [30]
JCVI_7830	Putative At2g46240	20	AT2G46240	*BAG6*	79.18	[29], [30]
JCVI_16615	Stress-induced protein sti1-like protein	20	AT4G12400	*HOP3*	41.91	[31]
JCVI_15301	Stress-induced protein sti1-like protein	20	AT4G12400	*HOP3*	23.72	[31]
JCVI_13423	Putative uncharacterized protein At4g21320/T6K22_50	20	AT4G21320	*Hsa32*	15.31	[33]
JCVI_1535	Bax inhibitor-like protein	20	AT5G47120	*BI-1*	11.80	[32]
JCVI_30402	Abscisic acid 8′-hydroxylase 4	26	AT3G19270	*CYP707A4*	43.44	
JCVI_24064	At5g37340	27	AT5G37340	*ZPR1 zinc-finger domain protein*	27.47	
JCVI_24249	Dehydration-responsive element-binding protein 2A	27	AT5G05410	*DREB2A*	22.58	[34]
EX057175	At5g37340	27	AT5G37340	*ZPR1 zinc-finger domain protein*	12.53	
CX193483	F2H15.10	29	AT1G17870	*EGY3*	57.37	[42]
ES265899	FtsH-like protein Pftf-like	29	AT5G15250	*FTSH6*	44.07	[43]
JCVI_5623	Putative uncharacterized protein T1P2.12	29	AT1G30070		18.55	
JCVI_29640	Ubiquitin	29	AT4G02890		13.20	
JCVI_20545	Protein-methionine-S-oxide reductase	29	AT4G25130	*MSRA4*	13.14	[41]
JCVI_6897	Elongation factor 1-beta 1	29	AT5G12110	*translation elongation factor EF1B*	10.77	
JCVI_15245	T24P13.19	29	AT1G26800		10.17	
JCVI_21312	Peptidylprolyl isomerase	31	AT5G48570	*ROF2*	289.16	[46]
JCVI_24281	Peptidylprolyl isomerase	31	AT5G48570	*ROF2*	141.09	[46]
JCVI_29636	Putative uncharacterized protein At3g10500	33	AT3G10500	*NAC053/NTL4*	19.87	[87]
JCVI_17638	Atfp6-like protein	34	AT5G66110		19.71	
JCVI_18266	F28G4.4 protein	34	AT1G72660		12.45	

a Locus name in the genome of *A. thaliana.*

b Fold change (FC) was expressed as the ratio of normalized expression of heat treatment to control.

In addition to *HSFs*, other transcription factors also participated in heat responses. For example, a homolog of *DREB2a* (JCVI_24249) was up-regulated by 22.6-fold (Tables 1, S3). *DREB2a* regulates plant thermotolerance in *Arabidopsis* by modulating the expression of *HsfA3* [34]. Other highly induced transcription factors included zinc-finger genes (JCVI_24064, EX057175 and JCVI_25058), MYB genes (*EPR1*, JCVI_19788 and EV135969) and one homeodomain gene (JCVI_3973) (Table S3). Of these genes, *ZAT12* (JCVI_25058) plays a central role in oxidative stress and cold signaling [35], [36], and *EPR1* mediates the *Arabidopsis* circadian clock [37]. However, their definitive functions in heat stress are still unknown. A heat stress-induced splice variant, *HSFA2-III*, regulates *HSFA2* transcription in *Arabidopsi*s, suggesting that alternative splicing is important for heat regulation [38]. In our study, five *serine/arginine-rich (SR)30*/*SR33* homologous genes involved in alternative splicing were obviously induced (Table S3). In *Arabidopsis*, pre-mRNAs of both *SR30* and *SR33* are alternatively spliced [39], [40], and *SR33* is regulated redundantly by *SR30* and *SR33* [40]. The splicing patterns of *SR30* can be altered under various stressful conditions, including high-light irradiation and salinity [39].

Heat stress may lead to damaged and unfolded proteins, which are harmful to plant cells. Several genes related to protein post-modification and degradation, including *EGY3* (CX193483), *FTSH6* (ES265899) and *MSRA4* (JCVI_20545), were obviously induced (Tables 1, S3). These three genes are closely connected to the chloroplast compartment (development, LCHII degradation and ROS eliminator) [41]–[43], in agreement with a previous report that the chloroplast and its functions are sensitive to heat stress [44]. Moreover, *SKP2B* (JCVI_9880) and *SGT1A* (JCVI_24968 and JCVI_18054), which may function in SCF (TIR1)-mediated protein degradation, were also highly induced (Table S3); a mutant of *SGT1A* has comprised thermotolerance in *Arabidopsis* [45].

Thirteen genes in other processes were also obviously up-regulated (>10-fold) in siliques (Table 1). Some of these notably induced genes were previously reported to be involved in heat regulation [46]–[48], such as *ROF2* (JCVI_21312 and JCVI_24281), *GolS1* (JCVI_26215 and JCVI_40980) and *MBF1c* (JCVI_802), which were classified into the categories of vesicle transport (Bin 31), synthesis of minor carbohydrate (Bin 3) and hormone signaling (Bin 17), respectively (Figure 3A and Tables 1, S3). Interestingly, two previously uncharacterized genes were up-regulated by more than 50-fold in siliques (Figure 3A and Tables 1, S3); these genes encoded ABA 8′-hydroxylase (*CYP707A4*, JCVI_30402) (Bin 26) and cytochrome bd ubiquinol oxidase (JCVI_33865) (Bin 9).

Taken together, our data showed that a large number of heat-stress related marker genes, including *HSF/HSP*, *DREB2a*, *ROF2*, *MBF1c* and *Hsa32*, were up-regulated in the SW and seeds simultaneously, suggesting that the heat treatment regimen we adopted was reliable and that a conserved mechanism operates during the heat response in different plants and organs. In contrast, few genes were down-regulated in both organs, suggesting that the repression of transcription during heat stress is a passive adaptive mechanism that is notably different between pods and seeds (see below, Figure 2). Furthermore, the newly identified transcripts further enriched the reservoir of heat-responsive genes.

### Identification of genes preferentially expressed in response to heat stress in the SW

In total, there were 411 genes preferentially up-regulated in the SW (group II) but not in seeds (group III) after heat treatment. These genes were distributed among 23 functional categories, exhibiting 2.0- to 18.9-fold changes (Figures 1C, 2, 3C and Table S1). Similar to group I, the top three most highly enriched categories were stress (48, 11.7%, Bin 20), RNA (43, 10.5%, Bin 27) and protein (40, 9.7%, Bin 29; Figure 3C and Table S1). One difference was that the number of genes in the transport category (22, 5.35%, Bin 34) was obviously increased (Figure 3C and Table S1).

Genes down-regulated by high temperature may cause impaired development in plants [49]. Compared with the number of up-regulated genes, more genes (514) were down-regulated in the SW. These genes were classified into 29 functional categories, and they exhibited less dramatic changes (2.1- to 3.5-fold; Figures 1C, 2 and 3D and Table S1). The top five categories with the most down-regulated genes included RNA (49, 9.5%, Bin 27), miscellaneous enzymes (40, 7.8%, Bin 26) and transport (31, 6.03%, Bin 34), followed by secondary metabolism (29, 5.6%, Bin 16) and protein (27, 5.3%, Bin 29). A relative small portion of genes involved in stress (22, 4.3%, Bin 20) were down-regulated compared with the up-regulated genes (Figure 3D and Table S1).

Compared with the up-regulated genes in siliques, some distinct genes were found to regulate RNA and protein. There were at least three transcription factors, including the zinc-finger gene *ZF1* (JCVI_27770) and the two ERF/AP2 family genes *ERF73* (JCVI_18164) and *RAP2.10*/*DEAR4* (JCVI_4906), that were significantly induced by 5- to 20-fold in the SW, while they were expressed at very low levels in seeds under normal and heat-stressed conditions (Table S5). These genes are negative regulators with other abiotic stress treatments, such as osmotic stress (*ZF1*) [50], oxidative stress (*ERF73*) [51] and plant defense and freezing stress responses (*DEAR1*, which belongs to the same subfamily as *DEAR4*) [52]. The disparity in responses to different stresses is worth further investigation. In addition, *ubiquitin-specific protease 18* (*UBP18*, JCVI_34965 and EV180578) and *senescence-associated gene 12* (*SAG12*, EV223498), which might participate in protein degradation, were also remarkably up-regulated by 10- to 15-fold (Tables 2, S5). The latter regulates developmental senescence in *Arabidopsis* and *B. napu*s [53] and is induced by a combination stress of heat and drought in tobacco [54].

**Table 2:** List of genes (*Hsf* and *Hsp* genes excluded) exhibiting more than 10-fold changes in expression only in the SW (group II) or seeds (group III).

Gene	Annotation	Bin	ATH^a^	Gene name	FC^b^	Reference
***Group II***						
EV199291	F15H11.1 protein	1	AT1G70760	*CRR23*	65.39	
JCVI_16753	Arabidopsis thaliana genomic DNA, chromosome 5, P1 clone:MKD15	16	AT5G23230	*NIC2*	17.34	
JCVI_2556	Putative myrosinase-binding protein 3	26	AT1G52030	*MBP1.2*	0.03	
JCVI_27770	Zinc-finger protein-1	27	AT5G67450	*ZF1*	16.20	[50]
JCVI_34965	Putative At1g24575	29	AT4G31670	*UBP18*	14.75	
EV180578	Putative At1g24575	29	AT4G31670	*UBP18*	14.24	
EV223498	Senescence-specific cysteine protease	29	AT5G45890	*SAG12*	10.27	[53], [54]
JCVI_25642	SGT1a	29	AT4G23570	*SGT1A*	10.09	[45]
JCVI_21265	F24J8.15 protein	30	AT1G21550		22.66	
JCVI_17979	Sugar transporter ERD6	34	AT1G08930	*EARLY RESPONSE TO DEHYDRATION 6 (ERD6)*	12.65	
JCVI_35394	YUP8H12R.2 protein	34	AT1G79360	*ORGANIC CATION/CARNITINE TRANSPORTER 2 (OCT2)*	12.07	
JCVI_26879	Male sterility 2-like protein	11	AT4G33790	*CER4*	0.04	[59]
JCVI_10648	Adenylyl-sulfate kinase 2,	14	AT4G39940	*APK2*	0.10	
EX132209	Adenylyl-sulfate kinase 2,	14	AT4G39940	*APK2*	0.10	
JCVI_6514	Putative uncharacterized protein	16	AT3G57010	*Calcium-dependent phosphotriesterase superfamily protein*	0.09	
H74709	Putative uncharacterized protein	16	AT3G57010	*Calcium-dependent phosphotriesterase superfamily protein*	0.10	
JCVI_1570	Putative uncharacterized protein F24I3.90	16	AT3G57010	*Calcium-dependent phosphotriesterase superfamily protein*	0.10	
ES911529	Protein At5g04380	17	AT5G04380	*S-adenosyl-L-methionine-dependent methyltransferases superfamily protein*	0.09	
JCVI_411	Myrosinase-binding protein related protein	26	AT1G52030	*MBP1.2*	0.03	
JCVI_445	Annexin D4	31	AT2G38750	*ANNAT4*	0.09	
***Group III***						
EE401951	Methylthioalkylmalate synthase precursor	16	AT5G23010	*MAM1*	23.82	[69]
JCVI_41485	Endoglucanase 19 precursor	26	AT4G11050	*GH9C3*	55.54	[66]
JCVI_34217	Glucuronosyl transferase-like protein	26	AT5G05900	*UDP-Glycosyltransferase*	11.14	
JCVI_3072	Putative uncharacterized protein At3g22640	33	AT3G22640	*PAP85*	14.53	[67]
JCVI_12895	Flavonol 3-O-glucosyltransferase-like protein	16	AT5G54010	*UDP-Glycosyltransferase superfamily protein*		

a Locus name in the genome of *A. thaliana.*

b Fold change (FC) was expressed as the ratio of normalized expression of heat treatment to control.

In heat-stressed SW, the most abundant down-regulated genes were related to RNA, but most exhibited 2- to 5-fold changes. The only exception was the mRNA for a bHLH transcription factor (AT2G46510, JCVI_4994), which was significantly down-regulated by 6.9-fold (Table S5). Other enriched down-regulated categories in the SW included multiple miscellaneous enzymes and secondary metabolism (Figure 3D, Table S1). Two myrosinase binding proteins (*MBP1.2*, JCVI_411 and JCVI_2556), a pyrophosphate-specific phosphatase (*PPsPaseI*, JCVI_13387) and three calcium-dependent phosphotriesterases (JCVI_1570, H74709 and JCVI_6514) exhibited the most striking declines (5.4-36.1-fold; Tables 2, S5).

### Heat stress affected several predominant metabolic pathways in the SW

The SW is the major photosynthetic organ after flowering, and its role becomes more predominant with the development of siliques [24]. With rapid seed development, the SW is important for metabolic processes and protection of the developing seeds. To understand the consequences of heat stimulus to the SW, we focused on several representative pathways.

Interestingly, a gene encoding a subunit of the chloroplast NAD(P)H dehydrogenase complex (*ORR23*, EV199291) was induced by 65.4-fold in the SW (Tables 2, S5), suggesting that the photosynthetic system of the treated siliques underwent adjustments upon the heat treatment. The SW also supplies the seed with energy, primarily during silique development [24]. Thus, genes with transporter activities could be important for seed development. In total, the expression of 53 transport-related genes was altered by heat stress in the SW (Tables S1, S5). Some of these genes were highly induced, such as the genes for sugar (*OCT2*, JCVI_35394; *ERD6*, JCVI_17979 and JCVI_8526; Table 2), water (aquaporins, JCVI_18822, JCVI_41889, JCVI_2150, EX065729, EV193206 and ES967441) and UDP-galactose transport (JCVI_31051, EX089759 and JCVI_15352) (Table S5). In contrast, the transcription of genes involved in a number of processes was down-regulated, including genes for ions (DY014304, DY014304, EH421119 and JCVI_19623), purine (EV193181, JCVI_1037, JCVI_1037 and EH421119), sucrose (*SUC1*, JCVI_13834, JCVI_14458 and EV181968), and sulfate (*SULTR3;1*, JCVI_16207 and EV134762) transport (Table S5). The expression of many genes for ABC transporters (JCVI_32851, JCVI_32851, JCVI_34086, JCVI_34487, JCVI_11077 and JCVI_38584) was also repressed (Table S5). These results suggested that up-regulated transporters primarily mediated the homeostasis of sugar and water, while down-regulated transporters played many roles. In *Arabidopsis,* the transport direction of vacuolar glucose (export or uptake) affects seed yields [55], [56]. Significant yield loss of *B. napus* exposed to high temperature [14] may possibly be related to glucose homeostasis regulated by the genes discovered in present study.

Up-regulation of cell wall modification enzymes has been reported to be a mechanism of heat regulation in *B. rapa* seedlings [17]. However, we observed the down-regulation of 26 genes encoding cell wall modification enzymes (Figure 3D and Table S1). Among these genes, *pectin methylesterase 35* (*PME35*, EV193389) and *EXPANSIN A5* (*EXPA5*, JCVI_18998), which regulate the mechanical strength of the supporting tissue or organ growth [57], [58], were reduced nearly 10-fold (Tables 2, S5). Additionally, the wax biosynthetic gene *CER4* (JCVI_26879, Bin 11), which is expressed in different organs including the outer layer of the silique except in the seed in *Arabidopsis* [59], was down-regulated by 26.7-fold in the SW after heat stress (Tables 2, S5), and this result corroborated the finding that temperature negatively affects the yield of wax [60]. The expression pattern of these genes indicated that the physical state of the SW as a mechanical supporter and protective layer might undergo a different response to heat treatment in seedlings.

**Figure 4:**
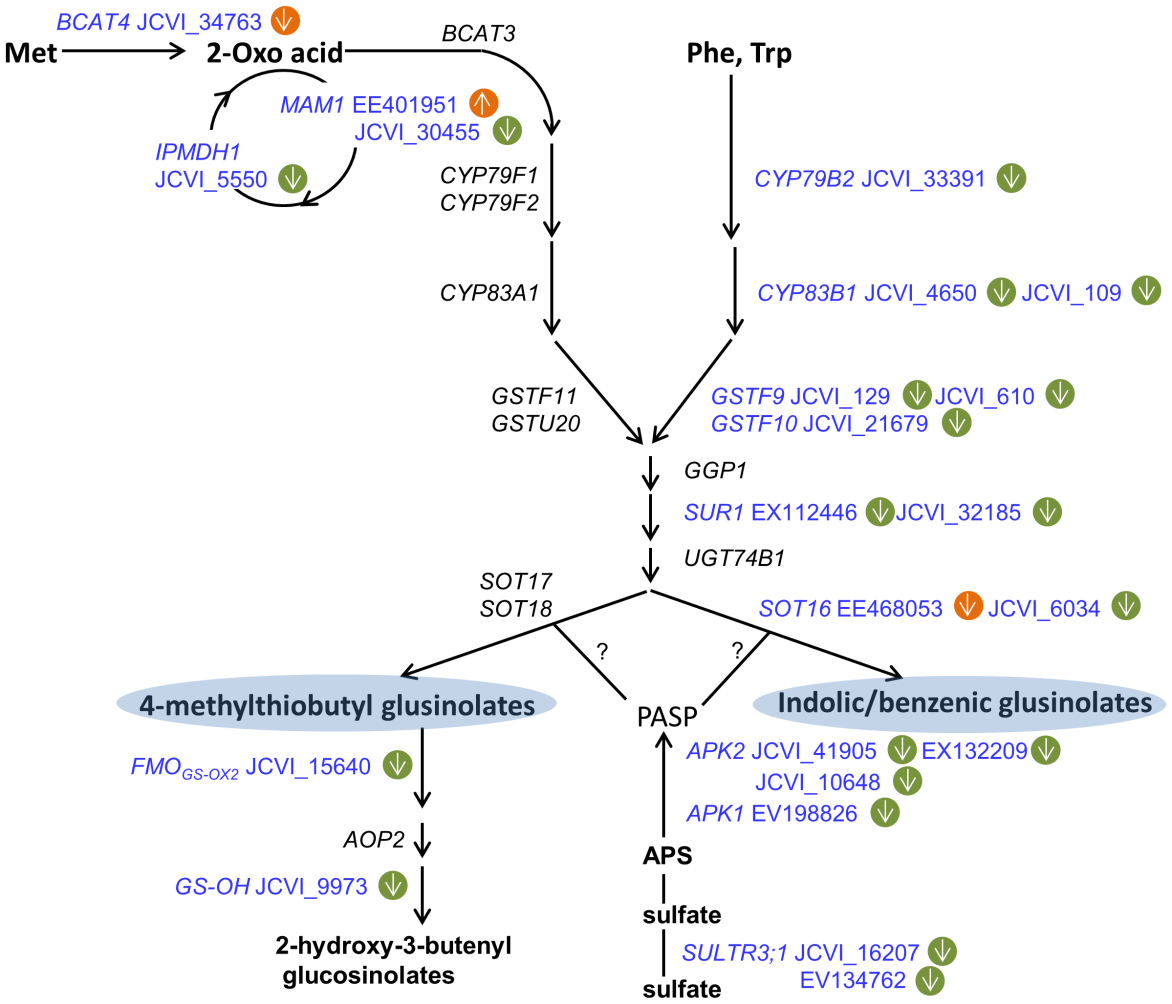
Differentially expressed genes involved in the aliphatic and indolic glucosinolate biosynthetic pathways. The green circle represents the SW, and the yellow circle indicates seeds. Arrows pointing up indicate increased expression; arrows pointing down indicate decreased expression. Abbreviations: BCAT, branched-chain amino acid aminotransferase; MAM, methylthioalkylmalate synthase; IPMDH, isopropylmalate dehydrogenase; CYP, cytochrome P450; GST, glutathione transferase; GGP1, γ-glutamyl peptidase 1; SUR1, C-S lyase; UGT, glucosyltransferase; SOT, sulfotransferase; FMO_GS-OX_, flavin monooxygenase; AOP2, 2-oxoglutarate-dependent dioxygenase; GS-OH, 2-oxo acid-dependent dioxygenase; APS, adenosine 5′-phosphosulfate; APK, APS kinase; SULTR, sulfate transporter; PAPS, 3′-phosphoadenosine-5′-phosphosulfate. This scheme was based on analyses by Maruyama-Nakashita et al. (2006) [97] and Sønderby et al. (2010) [62].

Glucosinolates are secondary metabolites that are unique in the Brassicales order including all Brassica species. The seed is the major compartment for their storage, while the synthesis of glucosinolates primarily occurs in leaves and the SW after flowering [61]. The expression of a subset of 10 transcripts (*FMO-GSOX2*, JCVI_15640; *CYP83B1*, JCVI_4650 and JCVI_109; *SOT16*, JCVI_6034; *MAM1*, JCVI_30455; *GS-OH*, JCVI_9973; *CYP79B2*, JCVI_33391; *GSTF9*, JCVI_129 and JCVI_610; *GSTF10*, JCVI_21679) in the categories of multiple miscellaneous enzymes (Bin 26) and secondary metabolism (Bin 16) involved in glucosinolate pathway [62] were reduced 3.2-fold on average. Especially, *CYP79B2* (JCVI_33391) in the indolic and benzenic pathways and *GS-OH* (JCVI_9973) in the aliphatic pathway were reduced by more than 5-fold (Figure 4 and Table S5), which suggested that glucosinolate synthesis was suppressed in the SW after heat stress. This hypothesis was also supported by another 10 transcripts in the glucosinolate biosynthetic pathway [62], [63] that were simultaneously reduced, including *BCAT4* (JCVI_34763), *IPMDH1* (JCVI_5550), *SUR1* (EX112446 and JCVI_32185), *APK1* (EV198826), *APK2* (JCVI_41905, EX132209 and JCVI_10648) and *SULTR* (JCVI_16207 and EV134762), which were designated into the categories of amino acid metabolism (Bin 13), S-assimilation (Bin 14) and transport (Bin 34) (Figure 4 and Table S5). Of these, three *APK2* transcripts were reduced by more than 10-fold (Tables 2, S5). However, the mechanism by which these enzymes affected plant performance under high temperatures was not clear.

Taken together, the genes involved in SW-specific metabolism indicate the presence of the following potential transcription features present in the heat-treated SW. First, the up-regulation of a subset of specific heat-response genes could prompt or rapidly induce a protective process upon heat shock; second, down-regulation of pathways may either serve as a passive adaptation or an active resistance/protection measure to save energy or reduce consumption, e.g., the down-regulated glucosinolate pathway. Such a result could provide clues for further metabolic profiling on these pathways.

### Identification of genes preferentially expressed in response to heat stress in seeds

Compared with the SW, fewer genes (325) were specifically up-regulated in seeds, and these genes were classified into 24 functional categories exhibiting 2.1- to 8.1-fold changes (Figures 1C, 2 and 3E and Table S1). There was approximately the same number (314) of down-regulated genes, which were assigned into 28 categories exhibiting 2.0- to 3.8-fold changes (Figures 1C, 2 and 3F and Table S1). The enriched categories for both up- and down-regulated genes followed a pattern similar to that in the SW (Figures 2, 3C-3F and Table S1).

One of the distinct features in response to heat stimulus in seeds was a set of seed-specific genes that were significantly induced. For example, out of 7 tested *HSFs*/*HSPs*, *HSFB1* (JCVI_5467) was strictly induced by nearly 6.8-fold in seeds (Table S6). Three other transcription factors, including the *ERF71* (JCVI_9816 and AM387841) involved in oxidative tolerance [64], the MYB-like transcription factor (EV176230) and the B-box zinc finger protein gene (*BBX25*, JCVI_15314), were induced by 5- to 10-fold (Table S6). Dramatic changes also occurred with genes potentially related to the development or quality of the seeds under normal or heat stress conditions. One enzyme, *GLYCOSYL HYDROLASE 9C3* (*GH9C3*; Bin 26), was dramatically induced 55.4-fold by heat stress from a very low initial level (Tables 2, S6). The inner integument of the ovule wall rapidly degrades at approximately 20 DAF in *B. napus* [65], and *GH9C3* belongs to a gene family involved in cell wall degradation [66]. The significantly induced expression of *GH9C3*, together with the induction of another cell wall modification gene, *XTH23* (Bin 10) (Tables 2, S6), may indicate that heat stress has a critical influence on cell wall degradation. Another gene homologous to *PAP85* (JCVI_3072) (Bin 33) encodes a vicilin-like seed storage protein [67] that is specifically expressed in late maturing-stage embryos in *Arabidopsis* [68]; this gene was induced 14.5-fold in the heat-stressed seeds (Tables 2, S6). These results indicated that these genes may function not only in developmental regulation but potentially also be involved in the enhancement of thermotolerance, specifically in the seed. Surprisingly, *MAM1*, which is responsible for methionine chain elongation during glucosinolate biosynthesis [69], was significantly induced by 23.8-fold from a very low basal level (Tables 2, S6). Previous study showed that elevated temperatures did not affect the glucosinolate concentration in *B. napus* [13]. Considering that gene clusters for glucosinolate biosynthesis were reduced in the SW (Figure 4), there might have a complementary response in the seeds to maintain glucosinolate content under heat stress.

### Heat stress affected seed-specific metabolic pathways

Lipid metabolism is the most active process in seeds during the seed-filling stage [70], [71]. Another feature of seed development at this time is seed coat color formation accompanied by the accumulation of phenolic compounds, which is highly correlated with *B. napus* oil quality [72].

**Figure 5:**
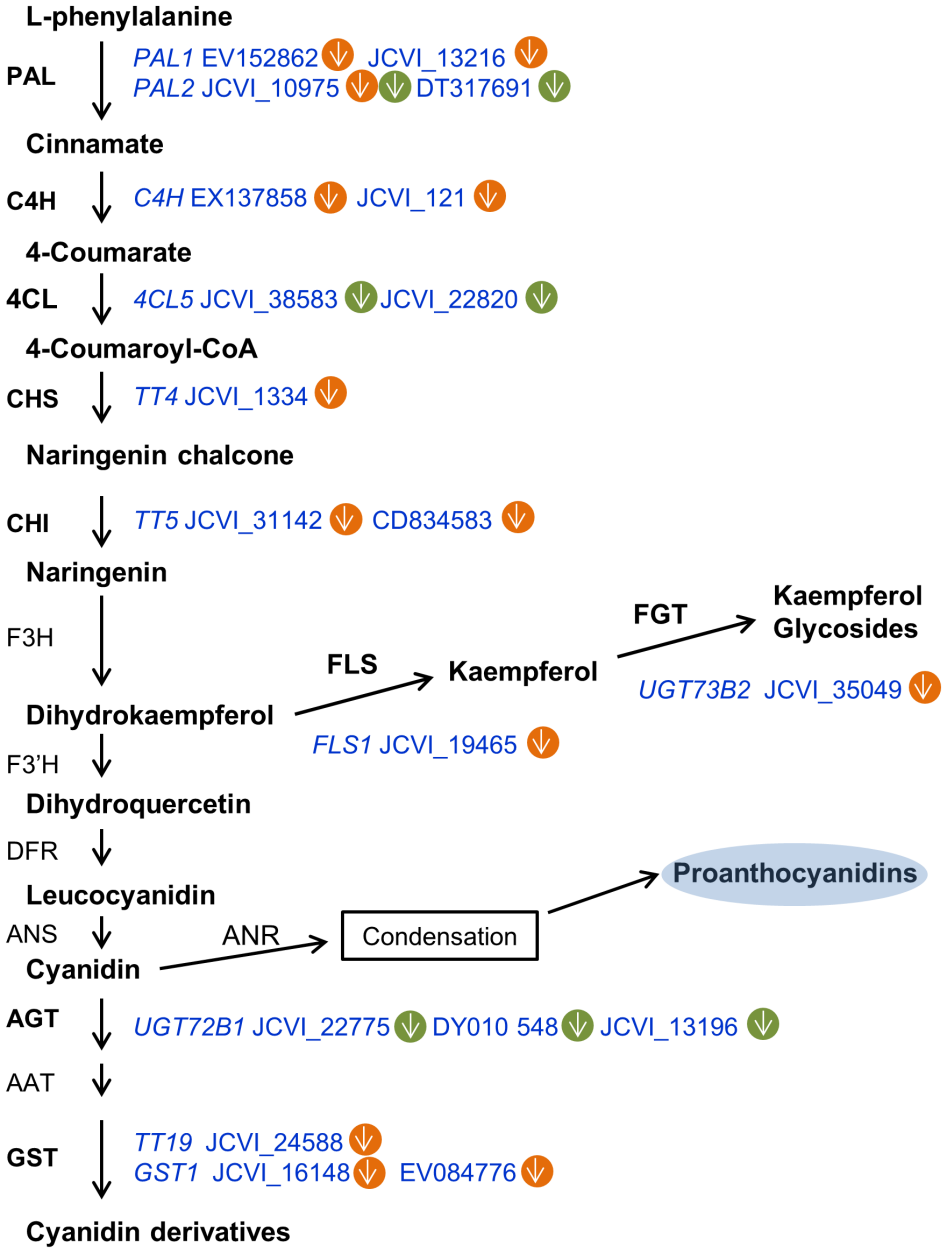
Differentially expressed genes involved in flavonoid and phenylpropanoid synthesis. The green circle represents SW, and the yellow circle indicates seed. Arrows pointing up show increased expression; arrows pointing down show decreased expressions. Abbreviations: PAL, phenylalanine ammonia lyase; C4H, cinnamate 4-hydroxylase; 4CL, 4-coumarate-CoA ligase; CHS, chalcone synthase; CHI, chalcone isomerase; F3H, flavanone 3-hydroxylase; F′3H, flavonoid 3′-hydroxylase; FLS, flavonol synthase; FGT, flavonol glycosyltransferase; DFR, dihydroflavonol 4-reductase; ANS, anthocyanidin synthase; AGT, anthocyani(di)n glycosyltransferase; AAT, anthocyanin acyltransferase; GST, glutathione S-transferase; ANR, anthocyanidin reductase. This scheme was based on the analysis by Lillo et al. (2008) [98].

Similar to the changes in the SW, some important metabolic processes in seeds were down-regulated upon heat treatment. A notable example was a cluster of 12 genes (*PAL1*, EV152862 and JCVI_13216; *C4H*, EX137858 and JCVI_121; *CHS*, JCVI_1334 and JCVI_31142; *CHI*, CD834583; *FLS1*, JCVI_19465; *UGT73B2*, JCVI_35049; *GST*, JCVI_24588, JCVI_16148 and EV084776) involved in flavonoid synthesis, which were simultaneously down-regulated (Figure 5 and Table S6). Among these, *PAL*, *C4H*, *TT4*, and *TT6* are key genes for seed coat pigmentation in *B. napus* [73]. Proanthocyanidins mainly accumulate in 15 to 35 DAF seeds of *B. napus* [65]. The depressed expression of the genes in this pathway suggested that high temperature might prevent pigmentation in seed coats. This result is consistent with the linear correlation between the reduction in color and temperature in yellow-seeded lines in *B. napus* [74]. However, the consequence of this change awaits for a further verification with metabolic analysis.

At 2–3 weeks after anthesis, storage oil begins to accumulate in young embryos by consumming nutrients from endosperm *in B. napus* [70], [75]. A group of 12 genes involved in lipid metabolism (Bin 11) were down-regulated on average by 3-fold in the seeds (20 DAF) after heat stress, including *GDPD2* (JCVI_21849 and JCVI_18449), *Acyl-ACP thioesterase* (JCVI_29193 and JCVI_16953), *fatty acid desaturases* (JCVI_24311; EV014782 and *ADS2*, JCVI_23874) and *fatty acid elongation 1* (*FAE1*, JCVI_22797) (Table S6). Previously, a transcriptome comparison between *B. napus* strains with high- and low-oleic acid contents detected the up-regulation of a gene similar to *Arabidopsis ADS1*; another gene homologous to *Arabidopsis ADS2* was down-regulated in the high-oleic acid strain [76]. In our study, the same expression pattern was observed for *ADS1* and *ADS2* in the heat-stressed seeds (Table S6). This result may explain the previous observation of increased oleic acid content after heat stress [14].

In *B. napus*, gibberellins (GAs) and ethylene are rich in developing siliques (mainly in seeds), and indole-3-acetic acid (IAA) maintains a steady-state level during the seed-filling stage [77]–[79]. These phytohormones participate in silique development and seed maturation in *Arabidopsis* [80]–[82]. In our study, most of the genes involved in hormone signaling were down-regulated upon heat treatment, but their expression patterns varied between the different hormone signaling pathways. Synthetic genes for ethylene (*2-oxoglutarate-dependent dioxygenase*, JCVI_33477; *ACO1*, EE436585) and GA (*GA20OX3*, JCVI_34330 and JCVI_17833) were all reduced in the seeds after heat stress (Table S6), and both of these gene sets regulate plant thermotolerance in *Arabidopsis* [83], [84]. Conversely, genes responsible for auxin synthesis (*GH3.3*, JCVI_2400), binding (*ABP1*, ES913545 and JCVI_8950) and transport (*ASA1*, JCVI_19120) were all induced in seeds (Table S6). Interestingly, auxin positively promotes hypocotyl elongation at high temperature [85]. These data suggest a complex interaction among ethylene, GAs and IAA in regulation of the seed development after heat stress.

Taken together, comparison of the gene expression profiles in seeds and the SW suggested that a subset of specific heat-responsive genes in seeds might constitute another layer of resistance to heat stress. For example, metabolic pathways, such as flavonoid synthesis, may be rapidly turned down to conserve energy for the hypersensitive reaction to heat damage.

### Approximately one-third of heat-responsive genes encoded proteins with unknown functions

**Figure 6:**
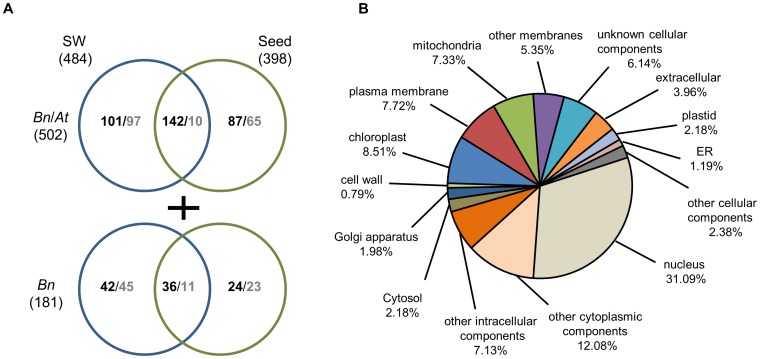
Differentially expressed genes with unknown functions in the heat-stressed SW and seed organs. (A) Venn diagram indicating the numbers of unknown genes conserved and not conserved with *Arabidopsis* that were detected in the SW or seeds. (B) Cellular components of 502 genes with unknown functions that were conserved with *Arabidopsis* based on GO analysis in TAIR (http://www.arabidopsis.org/).

Notably, we detected 484 (32.1%, 321 up and 163 down) and 398 (32.6%, 289 up and 109 down) genes with unknown functions in the SW and seeds, respectively (Figure 6A and Table S1). Among these genes, 502 (243 up and 107 down in the SW; 229 up and 75 down in seeds) were conserved in *A. thaliana*, and a subset of 181 transcripts (78 up and 56 down in the SW; 60 up and 34 down in seeds) with unknown functions was specific to *B. napus* (no homologs in *Arabidopsis*) (Figure 6A). Gene ontology (GO) analysis of cellular components for those 502 conserved genes indicated that approximately the same 7–8% of genes was localized in the chloroplast, plasma membrane and mitochondria. The proportion of genes targeted to the nucleus was higher (31%), suggesting that many unknown genes regulate the expression of other genes under heat stress (Figure 6B).

There were 142 genes up-regulated in both seeds and the SW with the fold change varying from 9.3- to 13.5-fold, accounting for 78.4–82.9% of the genes exhibiting more than 10-fold changes (Figures 3A, 6A and Table S7). In contrast, only 10 genes were simultaneously down-regulated, and they were altered by less than 3-fold in both organs (Figures 3B, 6A and Table S7), indicating that the down-regulated genes were differed between the SW and seeds.

There were 101 genes preferentially up-regulated by 4.2-fold in the SW, while 97 genes were down-regulated by 2.9-fold on average (Figures 3C-3D, 6A and Table S7). In seeds, 87 genes were up-regulated, and 65 genes were down-regulated to the same extent (3-fold on average) (Figures 3E-3F, 6A and Table S7).

Among the 181 genes specific to *B. napus*, the up-regulated genes exhibited more dramatic changes (13.6-, 11.3- and 5.8-fold in both organs, the SW alone and seeds alone, respectively) compared with the down-regulated genes (approximately 3.0-fold on average) (Figures 3A-3F). Only 18 mRNAs were annotated, and others were completely unknown based on a BLAST search against the NCBI non-redundant database (Table S8). Among the annotated genes, two genes encoding a splicing factor (DY004157) and a 17.5-kD class I heat shock protein (ES951677) were up-regulated by over 5-fold in both the SW and seeds, and another retrotransposon, Tto1 DNA, was enhanced only in seeds (Table S8). At least 13 unannotated genes were induced more than 10-fold. Of these genes, the EE438290 transcript was enhanced by more than 40-fold only in seeds (Table S8).

### Validation of microarray data by quantitative real-time (qRT)-PCR

**Figure 7:**
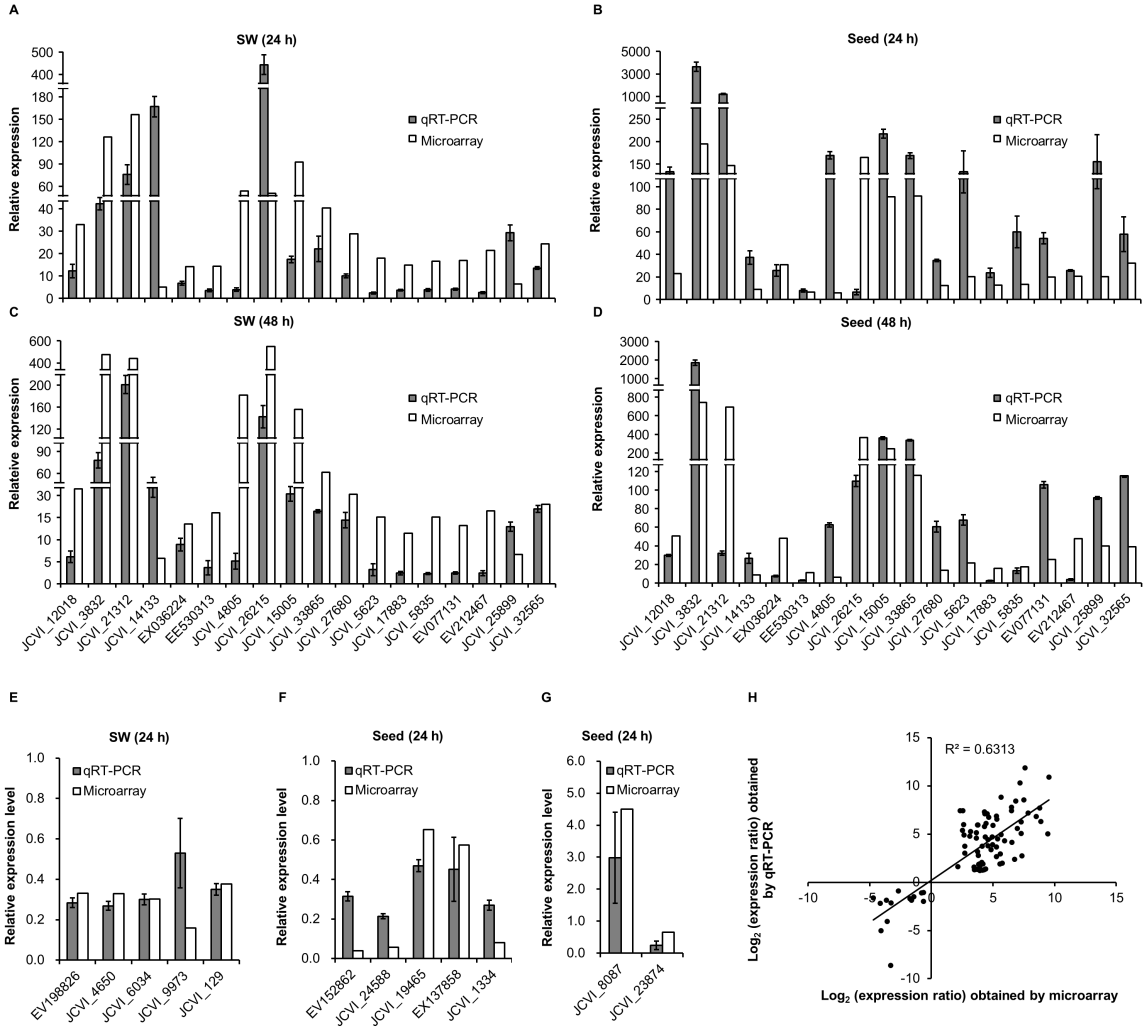
qRT-PCR validation of differential expression. Relative expression levels of 18 up-regulated genes were detected in the SW and seeds sampled at 24 h (A and B) and 48 h (C and D) after heat treatment. 10 down-regulated genes, of which 5 involving glucosinolate metabolism and another 5 associated with flavonoid synthesis were analyzed in the SW (E) and seeds (F) at 24 h after treatment respectively. Two genes may responsible for lipid synthesis were also detected in seeds (G). (H) Correlation of the gene expression ratios obtained from qRT-PCR and microarray data. The qRT-PCR log_2_ value of the expression ratio (y-axis) has been plotted against the value from the microarray (x-axis). The results of all the tested genes were listed in detail in Table S9. Data were collected from three biological replicates and three technical replicates for each sample.

Subsets of genes from the above categories or pathways were selected to validate the microarray data in heat-stressed SW or seeds by qRT-PCR. The relative expression levels measured by qRT-PCR were converted to change fold to enable direct comparison with microarray data (Figures 7A-7G, and Table S9). A total of 32 genes were analyzed and a linear regression analysis showed an overall correlation coefficient of R  =  0.795 between transcript levels assayed by the two detection systems, demonstrating the reliability of the microarray profiling (Figure 7H).

Among the 32 genes, 5 heat-inducible maker genes (*Hsp101-1* (JCVI_12018), *Hsp17.6-CII* (JCVI_3832), *GolS1* (JCVI_26215), *ROF2* (JCVI_21312) and *BAG6* (JCVI_15005)), together with additional 13 heat-stimulated genes with most of them belonging to unknown genes were from up-regulated genes. All of these genes were heat-induced with the similar tendency compared with the microarray data, although the fold changes may vary between the qRT-PCR and microarray measurements (Figures 7A-7D). In addition, 7 of the 18 up-regulated genes were also validated by reverse transcription (RT)-PCR for an extended time course (from 24 to 96 h after heat stress), and all of them displayed strong expressions after heat shock in SW and seeds than the controlled samples (Figure S2).The results suggested that the overall inductions may last for an extended recovery time after heat treatment, and the two time points used in this study were representative.

For down-regulated genes, we first validated a gene (ES911529) that was reduced only in heat-treated SW and another one (JCVI_12895) in seeds (Table S9). As expected, ES911529 was declined significantly in SW and could not be detected in seeds. Similarly, JCVI_12895 was hardly detected in SW and showed a reduction in seeds after heat stress. The results thus supported that the tendency of down-regulated expression with two methods were comparable. We then further validated 5 genes involved glucosinolate metabolism in SW (Figure 7E, Table S9) and 5 genes related to flavonoid synthesis in seeds (Figure 7F, Table S9). All of the genes were down-regulated with similar degrees compared with microarray data. In addition to the two pathways, two genes that were suggested to be correlated to the oleic acid content were also tested (Figure 7G). The result showed an up-regulation of *ADS1* (JCVI_8087) and a down-regulation of *ADS2* (JCVI_23874), which exactly matched the microarray data. Together, our qRT-PCR analysis further validated the microarray profiling under heat stress at the seed filling stage of oilseed rape.

### Identification of novel genes responsible for thermotolerance

*A. thaliana* belongs to the *Brassicaceae* family and diverged from *Brassica* 14–24 million years ago [86]. As a model plant with rich resources for functional genomics, *Arabidopsis* serves as a powerful tool to characterize the functions of genes identified in other *Brassica* species. To determine whether the heat-affected genes were responsible for plant thermotolerance, we selected eight genes from five different functional categories for further analysis (Table 3). Eight *Arabidopsis* T-DNA insertion mutant lines based on the candidate genes of *B. napus* were obtained from the Arabidopsis Biological Resource Center (ABRC). The homozygous lines of these mutants were isolated and confirmed by PCR analysis. RT-PCR showed that the expression of the selected gene in each mutant was completely abrogated (Figure S3). Some of these genes are known to participate in abiotic stress, such as *EPR1* in light rhythm [37], *NTL4* in ROS regulation during drought-induced senescence [87] and *SR45a* in alternative splicing under high light [88].

**Table 3:** Genes selected for mutant analysis.

Bin	No.	ATH a	Mutant lines	Gene	Annotation	Fold change b			
						SW (24 h)	SW (48 h)	Seed (24 h)	Seed (48 h)
3	*h23*	AT2G22240	SALK_101357	JCVI_14133	Myo-inositol-1-phosphate synthase (MIPS) 2	5.1	5.8	9.1	8.7
4	*h29*	AT2G36460	SALK_014964C	JCVI_15840	Fructose-bisphosphate aldolase (FBA) 6	9.4	16.9	4.6	6.6
27	*h15*	AT1G18330	SALK_047716C	JCVI_19788	Early-phytochrome responsive (EPR) 1	6	6	5.6	6.5
33	*h34*	AT3G10500	SALK_009578C	JCVI_29636	NAC transcription factor (NTL) 4	12.8	10.5	22.9	50.6
35	*h17*	AT3G12050	SALK_108314C	JCVI_2994	Aha1 domain-containing protein	7.8	6.5	6.8	6.4
35	*h25*	AT1G07350	CS853362	EV212467	Serine/arginine rich-like protein, SR45a	20.9	19.6	20.7	47.6
35	*h26*	AT1G03070	SALK_066103C	JCVI_25899	Bax inhibitor-1 family protein	6.2	6.7	20.2	39.9
35	*h38*	AT4G23493	SALK_131136C	EX036224	Putative uncharacterized protein	14.2	13.5	30.9	48.3

a Locus name in the genome of *A. thaliana*.

b Fold change was expressed as the ratio of normalized expression of heat treatment to control.

**Figure 8:**
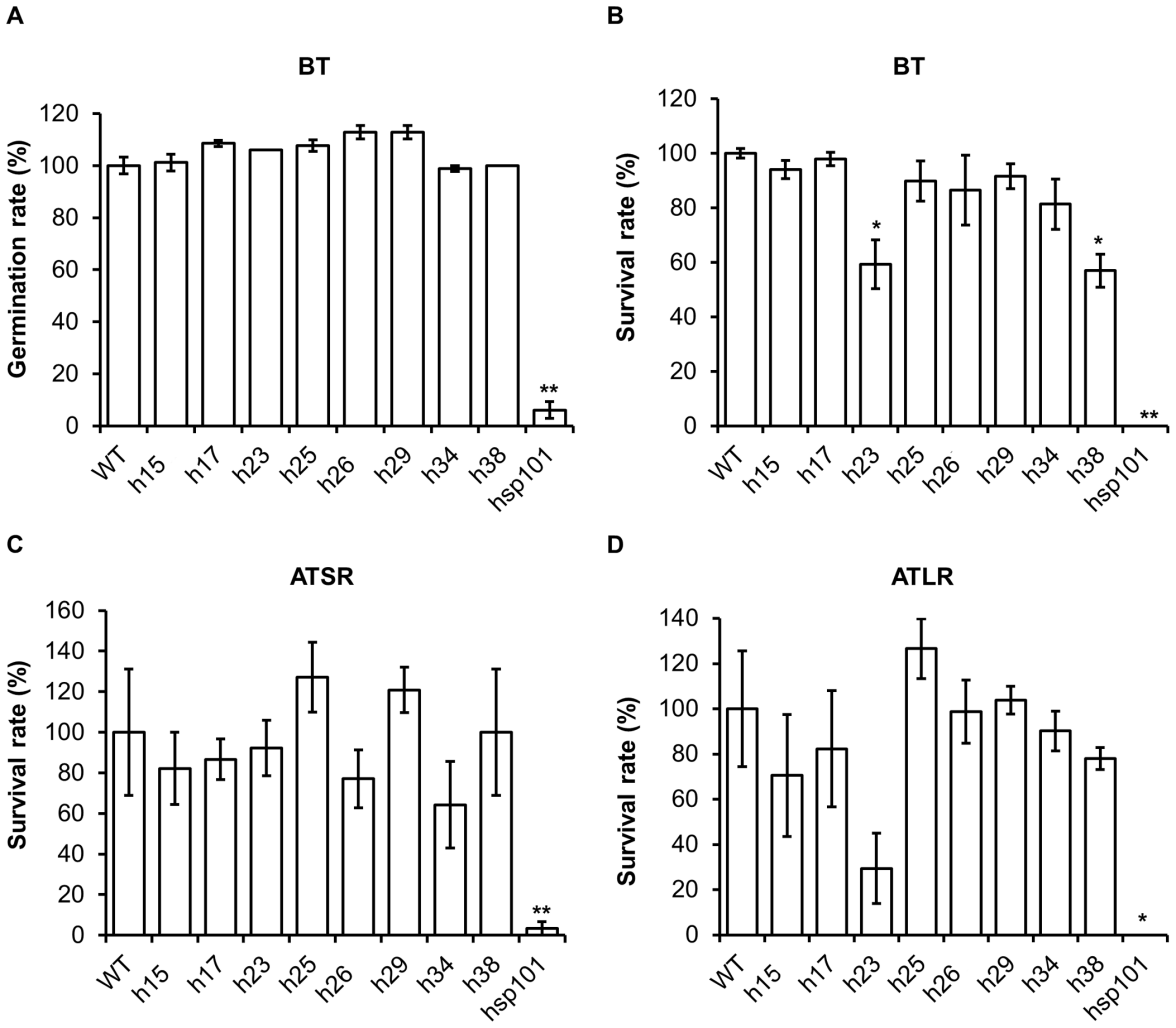
Heat stress treatment of mutant lines. (A) Germination rates for the BT assay. Seeds were treated at 45°C for 5 h immediately after vernalization at 4°C, and germination rates were measured after an additional 2 d. Survival rates for the BT assay at 45°C for 30 min (B), ATSR assay at 45°C for 150 min (C) and ATLR assay at 45°C for 1 h (D). 7-d-old seedlings were used in analyses and survival rates of each sample was measured at 7 dafter heat stress. Data were collected from three replicates, each with 30 seeds or 20 seedlings. * and ** indicate significant differences compared with WT plants for *P*<0.05 and *P*<0.01, respectively, as determined by a t-test.

**Figure 9:**
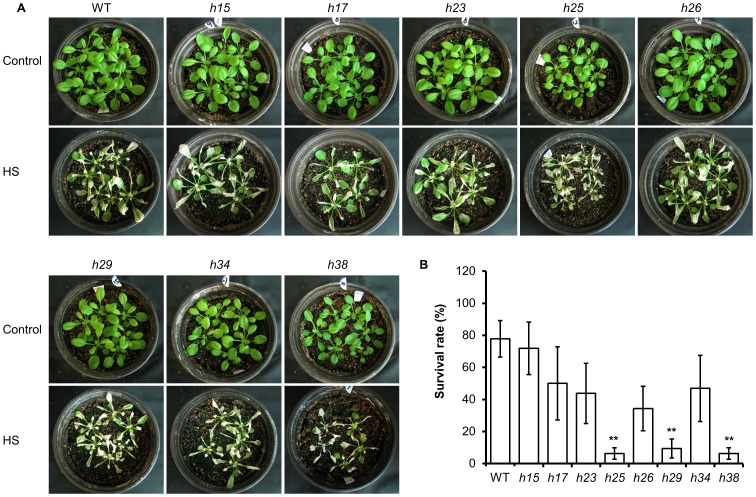
Heat stress tolerance of soil-based plants during continuous heat treatment. (A) Plants under control and heat stress (HS) conditions are shown in parallel. (B) Survival rates of mutant lines after continuous heat treatment. Data were collected at 3 weeks after heat stress in A from three independent replicates. ** indicates a significant difference compared with WT plants for *P*<0.01 based on a t-test.

To characterize the thermotolerance of the mutant plant lines, their seeds were treated at 45°C for 5 h. The germination rates of all the mutant lines were not significantly different from WT plants, but the positive control *hsp101* decreased to 6.1% of WT (Figure 8A). Because no significant difference in basal thermotolerance with seed was observed, severe heat stress (45°C for 30 min) was applied to 7-d-old seedlings grown under normal condition directly, or acclimated from a moderate heat stress treatment (38°C for 90 min) which was followed by either a short (2 h) or long recovery (2 d). Two mutant lines, *h23* and *h38*, exhibited reduced survival rates (59.3% and 56.9% of WT, respectively) (Figures 8B-8D), suggesting that the genes were involved in thermotolerance during the vegetative growth stage. We did not detect any difference in acquired thermotolerance between mutants and WT plants after both long and short recovery experiments. This result corroborated a previous study showing that mutants of *AT3G12050*, *MIPS* and *AT1G03070* exhibited no difference compared to WT plants in an acquired thermotolerance experiment with a long recovery [89]. We extended our experiment to mature plants grown under moderate high temperature for a long time period (38°C for 9 h). Under this condition, *h25*, *h29* and *h38* mutants were more sensitive than WT plants, with mostly withered leaves after a 3-d recovery from heat stress (Figure 9A) and significantly lower survival rates after 3 weeks (Figure 9B). Together, these results reflected the complexity of heat regulation mechanisms, which were specific to the development stage and dependent on treatment methods. Nevertheless, we identified novel genes involved in heat stress responses based on gene expression data.

## Materials and Methods

### Plant materials and growth conditions

Zhongshuang 9, a semi-winter *B. napus* variety developed at the Oil Crops Research Institute (CAAS, Wuhan 430062, China) was grown in a greenhouse at 23/18°C (day/night) and a relative humidity of 70% under a 13-h illumination time with a light density of 230–300 µEm^−2^s^−1^. At the three-leaf stage, seedlings were vernalized at 4°C for 14 d. Seedlings were returned to the greenhouse and transplanted to a larger pot with one seedling per pot. Plants were heat-treated during pod development as in Figure S1; siliques at 20 DAF were randomly sampled after a recovery time of 24 h and 48 h. The developing SW and seeds were separated and pooled as two organ samples. All samples were divided and used in parallel for cDNA chip hybridization and RT-PCR verification.

All of the *Arabidopsis* mutant lines were obtained from the ABRC. Homozygous lines of these mutants were identified by PCR analysis, and the accumulation of corresponding transcripts was detected by RT-PCR. Primers used for mutant verification are listed in Table S10. Plants were grown in a growth chamber with the conditions described in [90]. For the basal thermotolerance (BT) assay, seeds or 7-d-old seedlings grown on plates were heated at 45°C in a water bath for the indicated time. For the acquired thermotolerance (AT) assays, 7-d-old seedlings were first acclimated from 38°C for 90 min. For the acquired thermotolerance after short recovery (ATSR) assay, the plants were allowed to recover for 2 h at 22°C and then treated for 150 min at 45°C. For the acquired thermotolerance after long recovery (ATLR) assay, the plants were acclimated as described above, allowed to recover for 2 d at 22°C and then treated for 1 h at 45°C [33], [45]. For continuous moderate heat stress, 21- to 28-d-old plants grown in soil were exposed to a temperature of 38°C for 9 h in the dark and permitted to recover at 22°C for 3 d under continuous light conditions [91]. Three weeks later, plants that were still green and producing new leaves were scored as survived according to Larkindale et al. (2005) [92]. The data were expressed as the mean ± standard error (SE) (n = three biological replicates, 16 plants were analyzed for each replicate).

### RNA preparation and cDNA chip hybridization

Total RNA was extracted using a cetyltrimethylammonium bromide extraction method [93] and assessed by spectrophotometry and bioanalysis before proceeding to analysis with the Agilent Brassica 95k EST Microarray [25], developed by the John Innes Centre in collaboration with JCVI (J. Craig Venter Institute) and Cogenics. cDNA synthesis, labeling, hybridization, washing, scanning and data extraction were performed using established procedures for the analysis of eukaryotic RNA by the Cogenics Microarray Core Facility (Morrisville, NC, US).

### Microarray data analysis and quantification

Data were analyzed using a Perl script called BoaoAnalyzer (CapitalBio Corporation, Beijing, China) with per-spot and per-chip normalization. The LOWESS normalization method was used [94], and the threshold values of minimum and maximum signal intensities were 200 and 800, respectively. Signal intensities (relative mRNA prevalence) and signal detection calls were imported into Microsoft Excel for further analysis. Each probe set was manually assigned a consensus detection call in Microsoft Excel based on the BoaoAnalyzer detection calls of an RNA sample. Probe sets with signal detection calls of present (P, ≥800), absent (A, <200), or marginal (M, ≥200, <800) in 24-h and 48-h RNA samples were assigned consensus detection calls of PP, AA, or MM, respectively. Only probe sets with detection calls of PP (i.e., P in 24-h and 48-h RNA samples) were considered to represent a probe present in the SW or seeds. The Pearson correlation coefficient (r) for each pair of RNA samples was calculated using Microsoft Excel based on the signal intensities of the probe that was present. Genes with a ratio of treatment to control of more than 2 or less than 0.5 at both time points were selected. The microarray data were deposited in the public NCBI Gene Expression Omnibus database under the GEO accession number GSE57697.

### Gene functional classification and GO term enrichment analysis

Gene sets on the Brassica 95k EST microarray were classified into functional categories using MapMan 3.5.1 (http://mapman.gabipd.org/web/guest/mapman-version-3.5.1). mRNAs detected in the SW and seeds at the seed-filling stage in *B. napus* were analyzed for GO term enrichment with Blastx TAIR10 (http://www.arabidopsis.org/), InterProScan (http://www.ebi.ac.uk/InterProScan/) and agriGO (http://bioinfo.cau.edu.cn/agriGO/) [95]. For detecting significant enrichment of GO terms, we used the hypergeometric test methods and Benjamini-Yekutieli method [95].

### Validation of the microarray data

To validate the microarray data, 1 µg of total RNA was used to synthesize first strand cDNA with SuperScript III reverse transcriptase (Invitrogen) according to the manufacturer's instructions. For RT-PCR analysis, 0.2 µl of the cDNA was used in the subsequent PCR. All PCRs were performed in a total volume of 20 µl for 22–28 cycles under the following conditions: denaturation, 94°C, 30 s; annealing, 60°C, 30 s; extension, 72°C, 30 s. PCR amplification was performed with gene-specific primers using *BnActin* as a control (Table S10). Amplicons were visualized on a 1% agarose gel stained with ethidium bromide under UV light. qRT-PCR was performed as described by Li et al. [96]. The MyiQ sequence detection system (Bio-Rad) was used to detect products during quantitative real-time PCR by monitoring SYBR green fluorescent labeling of double-stranded DNA. The efficiency was normalized to the control gene *BnActin* with the primers listed in Table S10. The data were collected from three biological replicates, two technical replicates and expressed as the mean ± standard error (SE) (n =  three biological replicates). PCR conditions were as follows: one cycle of 95°C for 1 min; 40 cycles of DNA melting at 95°C for 30 s, DNA annealing at 55°C for 30 s and DNA extension at 72°C for 30 s; and a final extension of DNA at 72°C for 10 min.

## Supporting Information

Figure S1 **Heat treatment regimen for** ***B. napus*** **during pod development.** Plants underwent a stepped heat treatment during the daytime. For microarray analysis, the SW and seeds were sampled from 20 DAF siliques on these plants at 24 h or 48 h after heat stress.
(TIF)

Figure S2 **RT-PCR analysis of several putative heat-responsive genes during extended recovery time after treatment.** The SW and seeds were sampled after recovery times of 24 to 96 h following heat treatment as in Figure S1. H and C indicate heat-stressed and control samples, respectively.
(TIF)

Figure S3 **Summary of homozygous mutants tested for heat stress phenotypes.** The triangles indicate the locations of T-DNA insertions. RNA samples were purified from leaves for *h29*, *h34*, and *h25*; flowers for *h15a, h17* and *h26*; siliques for *h23* and roots for *h38*. *GSPs* is the abbreviation for gene specific primers, which are listed in Table S10.
(TIF)

Table S1 **Identification of functional categories that are enriched or reduced in the SW and/or seeds after heat stress during the seed-filling stage in** ***B. napus*** **.**
(XLSX)

Table S2 **Gene information corresponding to the heat map in** **Figure 2** **.**
(XLSX)

Table S3 **Genes that were differentially expressed (fold-change ≥2 or ≤0.5) in both the SW and seeds.**
(XLSX)

Table S4 ***HSF*** **/** ***HSP*** **genes that were significantly affected in heat-stressed SW/seeds.**
(XLSX)

Table S5 **Genes that were preferentially expressed (fold-change ≥2 or ≤0.5) in the SW.**
(XLSX)

Table S6 **Genes that were preferentially expressed (fold-change ≥2 or ≤0.5) in seeds.**
(XLSX)

Table S7 **Genes with unknown functions (conserved with** ***A. thaliana*** **) that were differentially expressed (fold-change ≥2 or ≤0.5) in the SW or seeds.**
(XLSX)

Table S8 **Genes with unknown functions (not conserved with** ***A. thaliana*** **) that were differentially expressed (fold-change ≥2 or ≤0.5) in the SW or seeds.**
(XLSX)

Table S9 **Validation of microarray data by qRT-PCR.**
(XLSX)

Table S10 **Primers used in this study.**
(XLSX)
