# Supplementary figures and images for: Identification of Heat Responsive Genes in Brassica napus Siliques at the Seed-Filling Stage through Transcriptional Profiling
Source: PLoS One. 2014 Jul 11;9(7):e101914. doi: 10.1371/journal.pone.0101914 (PMC4094393; doi:10.1371/journal.pone.0101914)

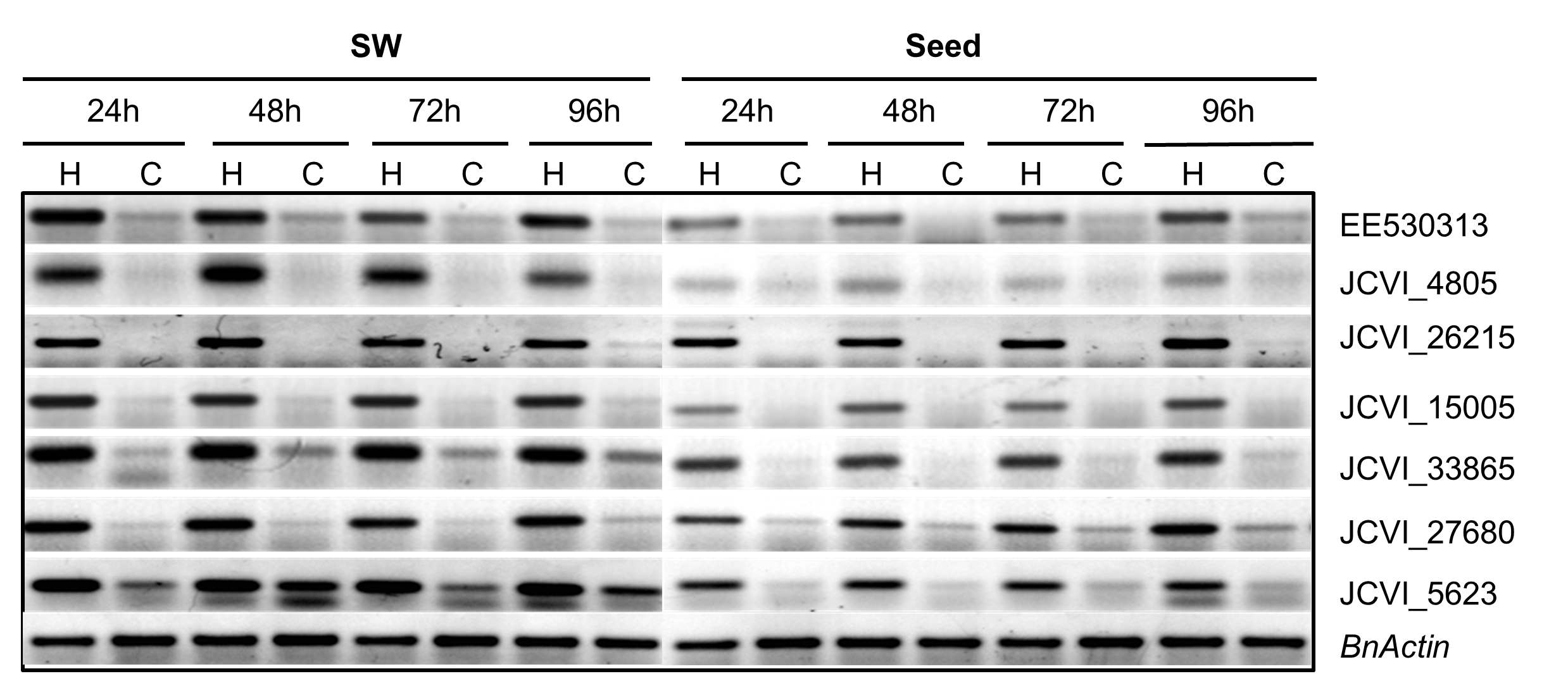

Supplement: Figure S2 — RT-PCR analysis of several putative heat-responsive genes during extended recovery time after treatment. The SW and seeds were sampled after recovery times of 24 to 96 h following heat treatment as in Figure S1. H and C indicate heat-stressed and control samples, respectively. (TIF) [file pone.0101914.s002.tif]
